# 199. Implementation of Novel Point-of-Care Hepatitis C RNA Platform and Clinical Characteristics of Treatment in Persons Experiencing Homelessness in Detroit, Michigan

**DOI:** 10.1093/ofid/ofaf695.071

**Published:** 2026-01-11

**Authors:** Kyle G Crooker, Yasmeen Mann, Michael Garcia, Mariia Numi, Brandon Ho, Richard Bryce, Marcus Zervos, Shaina Shetty, Seema Joshi

**Affiliations:** Henry Ford Hospital, Detroit, MI; Henry Ford Hospital, Detroit, MI; Henry Ford Hospital, Detroit, MI; Henry Ford Hospital, Detroit, MI; Henry Ford Hospital, Detroit, MI; Henry Ford Hospital, Detroit, MI; Henry Ford Hospital, Detroit, MI; CHASS Center, Detroit, Michigan; Henry Ford Hospital, Detroit, MI

## Abstract

**Background:**

Persons experiencing homelessness (PEH) have disproportionate rates of chronic hepatitis C virus (HCV) infection and face many barriers to care and follow up. Point-of-care (POC) testing attempts to minimize these barriers; however, traditional POC HCV antibody testing identifies exposure but not active infection. This limits timely diagnosis and treatment. The Cepheid POC HCV RNA test (Xpert^®^ HCV) allows for rapid detection of active HCV infection. Here we describe Xpert HCV utilization and HCV severity assessment for a test to treat model through street medicine with PEH.

**Figure 1:**
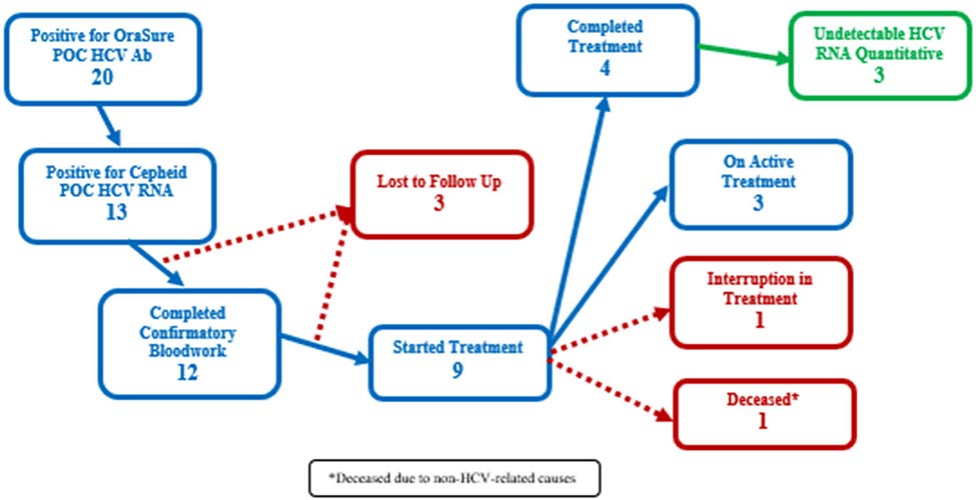
Flow Diagram of Patients with Hepatitis C Virus Infection

**Table 1:**
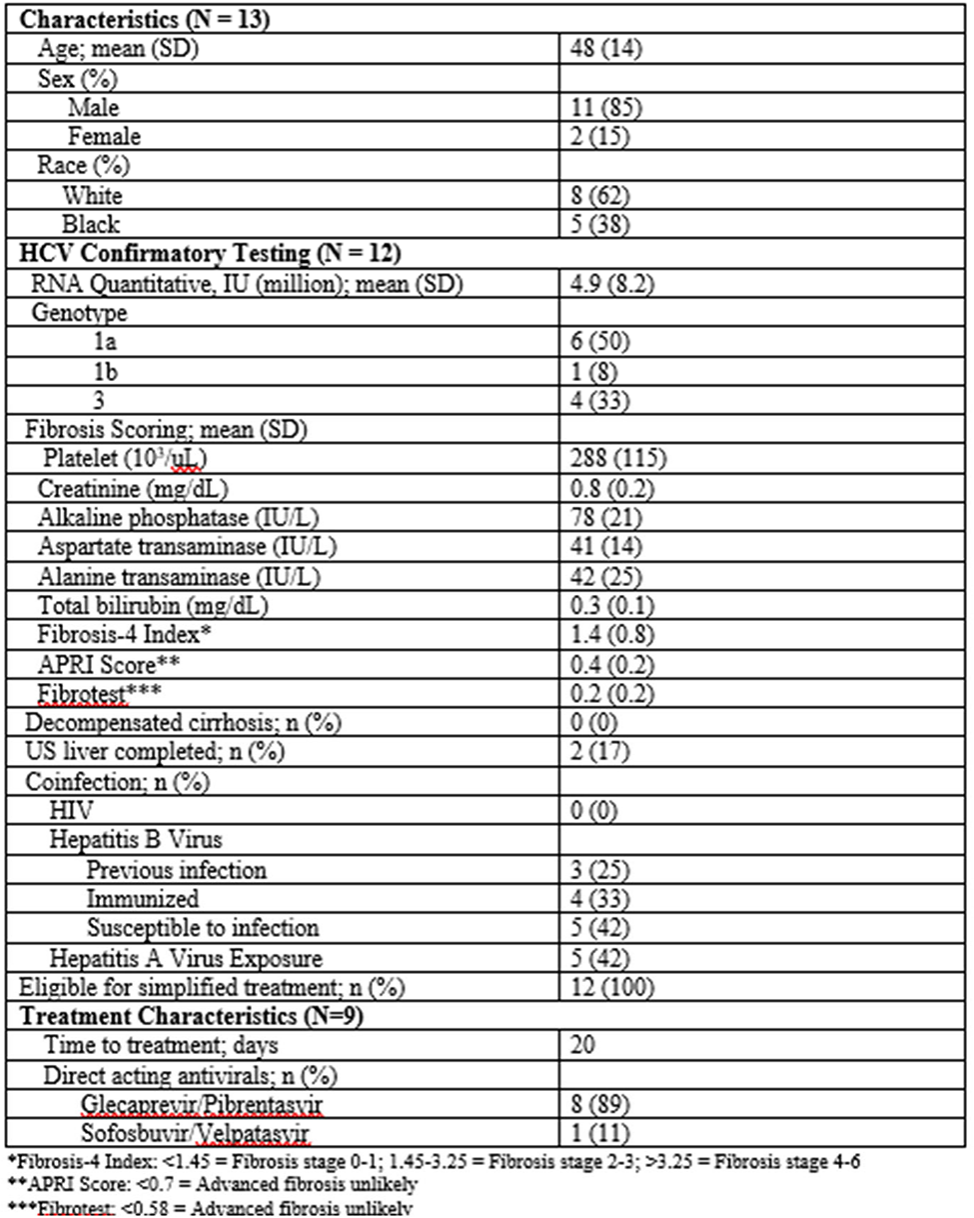
Patient characteristics, laboratory data and treatment characteristics of individuals with HCV

**Methods:**

From November 2024 to February 2025, weekly street medicine runs were conducted among PEH. Using finger stick, 250 µL of blood was collected for both the Orasure POC HCV antibody and Xpert HCV tests. Samples were run on the Cepheid POC Xpress system with qualitative results reported as detected or not detected for HCV RNA. Individuals with detectable HCV RNA underwent additional testing to determine HCV RNA quantitative value, genotype, and eligibility for simplified treatment. Demographic data and HCV risk factors were also obtained. Candidates for simplified treatment with direct-acting antivirals (DAAs) were delivered medications directly on a weekly or biweekly basis along with medication adherence outreach. HCV RNA quantitative data was obtained at end of treatment (EOT).

**Results:**

Twenty patients tested positive for HCV antibody and 13 tested positive for HCV RNA. Twelve patients were followed up with additional bloodwork. All patients were eligible for simplified treatment based on standard guidelines. No coinfections with HIV or HBV were identified. Nine patients received treatment, four completed treatment and three achieved undetectable HCV RNA at EOT (Figure 2). Demographic characteristics and HCV severity indicators are summarized in Table 1. The average time from test to treatment was 20 days.

**Conclusion:**

This study demonstrates that most patients are eligible for simplified treatment with DAAs, and the use of a POC HCV RNA platform enables rapid diagnosis and initiation of treatment in PEH. Integrating POC HCV RNA testing for marginalized populations allows for effective engagement and treatment to advance efforts in HCV elimination.

**Disclosures:**

All Authors: No reported disclosures

